# Assessment of Visual Acuity Outcomes in Ocular Syphilis Treated With Adjunctive Topical or Oral Steroids

**DOI:** 10.7759/cureus.41395

**Published:** 2023-07-05

**Authors:** Rhys Ishihara, Seth Buscho, Hannah J Yu, Praveena K Gupta

**Affiliations:** 1 Department of Ophthalmology and Visual Sciences, University of Texas Medical Branch, Galveston, USA

**Keywords:** adjunctive steroids, treponema pallidum, systemic steroids, topical steroids, ocular syphilis, syphilis

## Abstract

Purpose: There is no consensus surrounding adjunctive steroid use in the treatment of ocular syphilis. We evaluated clinical outcomes of patients with ocular syphilis who were treated with penicillin plus either topical or oral steroids.

Methods: Nine male patients aged 26 to 72 years with a diagnosis of ocular syphilis were retrospectively identified (18 eyes). All patients were treated with penicillin and adjunctive topical or oral steroids. Visual acuity reported as the logarithm of the minimum angle of resolution (logMAR) and slit lamp findings were documented at presentation, short-term follow-up (<7 days after initiating therapy), and long-term follow-up (day 7+). Visual acuity outcomes were compared between eyes treated with topical versus oral steroids as well as eyes treated simultaneously with adjunctive steroids and penicillin versus patients treated with steroids after penicillin.

Results: At short-term follow-up, the mean logMAR (SD, Snellen fraction) visual acuity for eyes treated with topical steroids 0.93 (0.53, 20/170) was significantly lower than that for the oral steroid group 0.23 (0.09, 20/110; p=0.0075). Similarly, at long-term follow-up, the topical steroid group had a significantly lower visual acuity of 0.75 (20/112) compared to a visual acuity of 0.07 (20/25) for the oral steroid group (p=0.0022). Moreover, the oral steroid group displayed significant improvement in visual acuity at long-term follow-up compared to baseline while the topical steroid group did not demonstrate the same effect (p=0.0406 and p=0.5945, respectively). Initiation of steroid treatment simultaneously with penicillin did not result in better visual acuity than delayed steroid treatment (p>0.05).

Conclusions: Steroids are an effective adjunctive treatment for patients with ocular syphilis. Oral steroids may be superior to topical steroids for improving visual function, especially in patients with a severe inflammatory component. Patients treated with oral or topical steroids and penicillin simultaneously did not demonstrate better visual acuity outcomes than patients treated with oral or topical steroids after penicillin was initiated.

## Introduction

Syphilis is a sexually transmitted infection caused by the spirochete, *Treponema pallidum*. The incidence of syphilis has greatly decreased since 1940 and reached an all-time low in 2001 at 2.1 cases per 100,000 people. Unfortunately, the incidence of syphilis has risen yearly since 2001 to reach a reported incidence of 10.8 per 100,000 people in 2018 [1]. It is estimated that 41.6% of individuals afflicted by ocular syphilis have a concomitant HIV infection [2]. Syphilis infection is notoriously difficult to diagnose as it may mimic many other pathologies and present in various stages. Primary syphilis is defined by a painless chancre at the site of infection, usually genital, which can last from two to eight weeks and subsequently progresses to secondary syphilis. In this stage, people may have systemic symptoms such as fever, chills, malaise, and desquamating rashes. Secondary syphilis may affect the eyes in many forms, including keratitis, iridocyclitis, episcleritis, and scleritis [3,4]. Neurosyphilis can occur at any time after secondary syphilis and is characterized by meningitis, tabes dorsalis, and general paralysis. Patients with neurosyphilis may have additional ocular findings, such as posterior or pan uveitis, optic neuritis, and acute syphilitic posterior placoid chorioretinitis [5,6].

Currently, the gold standard treatment for neurosyphilis is 3-4 million units of aqueous crystalline penicillin G intravenously every four hours for 10 to 14 days [7]. For ocular syphilis, however, standardized treatment is not as well-defined, likely due to the wide array of ophthalmic manifestations and their severity. Many different ophthalmologic treatment guidelines, including the Basic and Clinical Science Course and the American Academy of Ophthalmology, recommend adjunctive treatment with oral or topical steroids for uveitis secondary to ocular syphilis [8]. However, these guidelines do not include specifics regarding the initiation of steroid usage or the length of the treatment [5]. Also, the CDC does not provide concrete guidelines on the usage of adjunctive steroids for treatment of ocular syphilis [5,7]. This gap in the literature surrounding timely intervention of steroid use in the treatment of ocular syphilis has led to compromised visual recovery [9].

Furthermore, current ophthalmology literature has not come to a consensus on the use of topical versus oral steroids for uveitis secondary to ocular syphilis [9]. In the last several decades, only few reports have been published detailing topical steroid use in uveitis associated with ocular syphilis; however, those mainly consisted of case series or small cohort studies [4,6,9]. Additionally, some clinicians are hesitant to use steroids for ocular syphilis due to the high prevalence of concomitant HIV/AIDS and fear of exacerbating the infection. This potential side effect has yet to be thoroughly studied as evidence is primarily based on published case reports [10,11]. Therefore, current guidelines detailing the role of adjunctive steroids, both topical and systemic, in treating ocular syphilis are insufficient, indicating the need for further retrospective and prospective studies.

In this retrospective study, topical versus systemic adjunctive steroid use was evaluated among patients diagnosed with bilateral ocular syphilis. Secondary aims of this study were to identify common ophthalmic manifestations of ocular syphilis and evaluate whether the initiation timepoint of steroids altered visual outcomes for ocular syphilis.

## Materials and methods

Patient identification

This retrospective study was conducted at a tertiary medical center in Galveston, Texas. All protocols were approved by the University of Texas Medical Branch (UTMB) Institution Review Board (IRB). Patient data were collected and maintained in accordance with Health Insurance Portability and Accountability Act guidelines. Due to the retrospective nature of the study, no patient consent was required.

**Figure 1:**
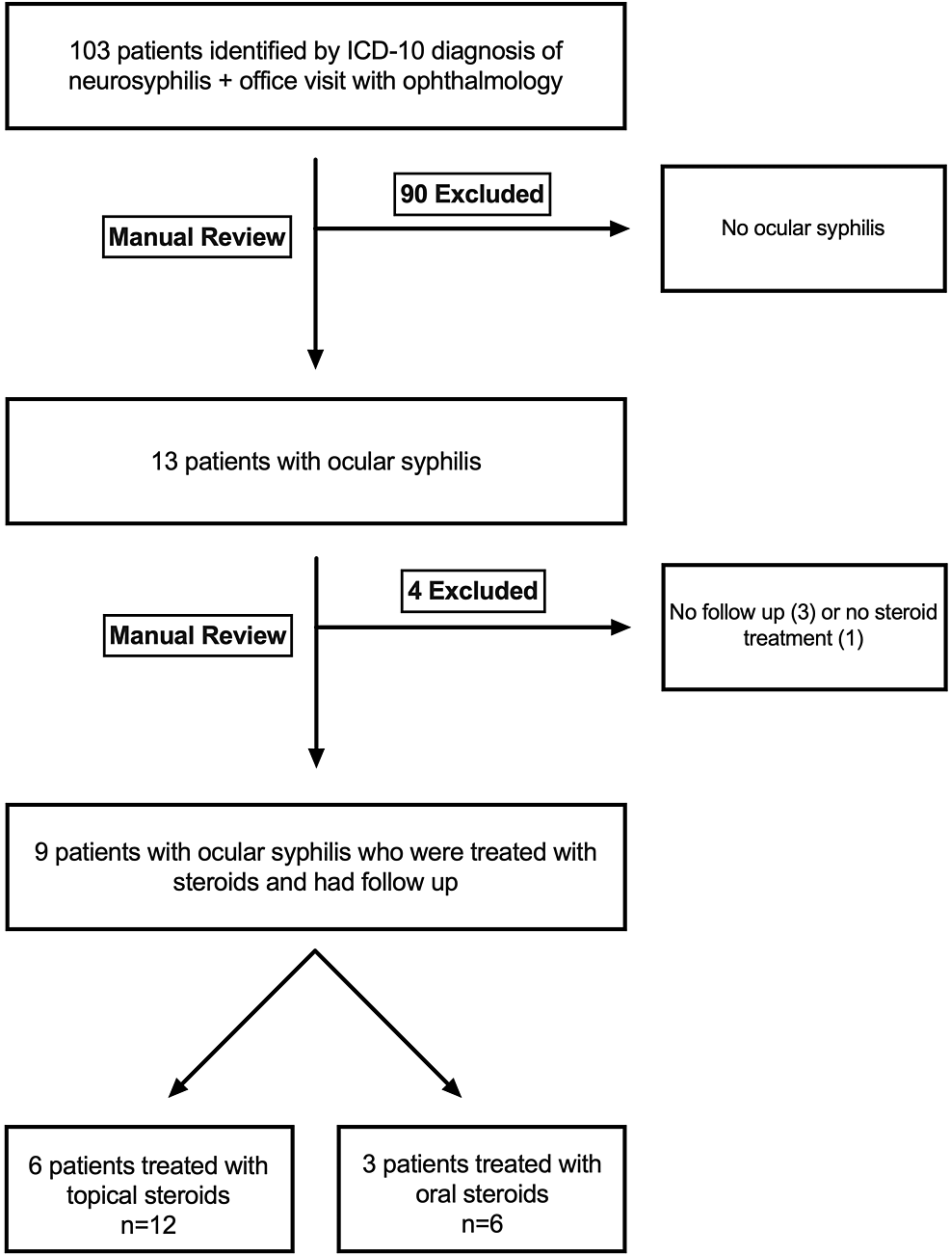
Subject acquisition Flowchart demonstrating how nine patients with bilateral ocular syphilis (18 eyes) were identified from the Epic database after manual exclusion for patients without ocular syphilis, treatment with steroids, or inadequate follow-up.

One hundred and three patients aged 18-100 years were identified from the Epic electronic medical records database with a diagnosis of neurosyphilis (ICD-10 code A52.19) and at least one ophthalmology examination between 01/01/2015 and 01/01/2021. Patients were included in the study if they were treated with penicillin and adjunctive steroids and had at least one follow-up with an ophthalmologist after treatment. Patients were excluded if they had a prior history of autoimmune disease, episode of undifferentiated uveitis, or history of uveitis secondary to another infectious cause (i.e., tuberculosis, toxoplasmosis, cytomegalovirus). The charts of the 103 patients identified were subsequently manually surveyed to confirm a diagnosis of ocular syphilis as defined by a positive syphilis test by a positive rapid plasma reagin (RPR) test, venereal disease research laboratory (VDRL) test, and/or positive treponemal IgG/IgM and a presentation of ocular syphilis with intraocular inflammation (Figure 1).

Data collected

Basic demographic information for all patients was compiled including age, sex, and race, as well as syphilis serologies and HIV status. Treatment protocols for antibiotics and steroids including length of treatment, route of administration, and dosage were also documented. The date at which the patient presented to the hospital, the date of diagnosis, and the date of discharge were recorded. Outcome measures for this study included short- and long-term best-corrected visual acuity (BCVA) and slit lamp findings assessed at baseline and at the time of discharge from the hospital or first clinical follow-up after discharge. Short-term BCVA was defined as BCVA measurements within six days of treatment while long-term outcomes were defined as BCVA at seven or more days post-treatment. All BCVA measurements were converted to the logarithm of the minimum angle of resolution (logMAR) for statistical analysis.

Statistical analysis

The mean and standard error of the mean were calculated and reported for each group. A student's t-test or analysis of variance with Tukey’s post hoc multiple comparison test was conducted using the GraphPad Prism program (GraphPad Software Inc., La Jolla, CA), and a P-value of <0.05 was considered statistically significant.

## Results

Demographics and serologies

Among the 13 patients identified with a diagnosis of ocular syphilis, we included 18 eyes from nine subjects who received topical or oral steroids for the treatment of ocular syphilis. The patients were all male and between the ages of 26 and 72 years. Patient demographic information and serologies are described in Table 1. Among the eight patients with positive RPR serologies, their titers ranged from 1:4 to 1:1024 with a mean (SD) RPR denominator of 230.p5 (342.5). The one patient who had a negative RPR had a positive fluorescent treponemal antibody test absorption test (FTA-ABS) with no prior history of syphilis, indicative of late-stage syphilis. There were five patients with positive HIV status, all of whom were immunocompetent with stage 1 HIV and a mean (SD) CD4+ count of 822.0 (350.8).

**Table 1:** Demographics and serologies of the included patients *: mean ± standard deviation (SD) of the nine patients; †: N (%) of the nine patients; RPR: Rapid plasma reagin

Demographics	
Age	40.44 ± 14.77*
Sex (male)	9 (100)^†^
Race (White)	6 (66.7)^†^
Race (Hispanic)	2 (22.2)^†^
Race (Black)	1 (11.1)^†^
Serologies	
Positive RPR status	8 (77.78)^†^
Positive serology but negative RPR	1 (22.2)^†^
Prior history of syphilis	1 (11.1)^†^
HIV status	5 (55.6)^†^

Treatment protocol

A total of six patients (12 eyes) were treated with penicillin and topical steroids (1.0% prednisolone acetate), and three patients (six eyes) were treated with penicillin and oral steroids. The treatment protocol was compared between the topical and oral steroid groups and was found not to be statistically significant (Table 2). All patients received intravenous (IV) penicillin within three days of a diagnosis of ocular syphilis and were treated for 7 to 14 days. The most frequent length of penicillin therapy was 14 days. The mean (SD) number of days between diagnosis and penicillin initiation was 1.13 (1.13) days and the mean (SD) duration of antibiotic treatment was 11.63 (2.72) days. The timepoint and length were similar for steroids. Steroids were initiated at the latest eight days after the diagnosis of ocular syphilis was made. The mean (SD) number of days between a diagnosis of ocular syphilis and initiation of steroids was 2.333 (3.20), and the mean (SD) length of steroid treatment was 18.56 (7.18) days.

**Table 2:** Treatment protocol for topical versus oral steroid groups N: Number of eyes; SD: standard deviation; P-value from Student’s t-test

	Topical mean ± SD N=12	Oral mean ± SD N=6	P-value
Days between presentation and antibiotic initiation	1.20 ± 1.30	1.00 ± 1.00	0.8286
Length of antibiotic regimen (days)	11.80 ± 3.19	11.33 ± 2.31	0.8345
Days between presentation and steroid initiation	1.33 ± 3.27	4.33 ± 2.31	0.2033
Length of steroid regimen (days)	17.17 ± 7.65	21.33 ± 6.51	0.4487

Baseline clinical characteristics

To assess common presentations of ocular syphilis, baseline slit lamp findings were recorded for all patients (Table 3). The most common ocular manifestation was panuveitis, which occurred in 66.7% of eyes. The second most frequent manifestation was optic neuritis followed by posterior uveitis and acute syphilitic posterior placoid chorioretinitis, occurring in 44.4% and 22.2% of patients, respectively. The least common presentation among our patient population was the Argyll Robertson pupil which was recorded in only one eye.

**Table 3:** Baseline slit lamp exam findings for 18 eyes N: Number of eyes

Clinical Characteristic	Total N (% of total patients)	Topical Steroids N (% of topical steroids patients)	Oral Steroids N (% of oral steroids patients)
Anterior uveitis	2 (11.1)	2 (16.7)	0 (0.0)
Posterior uveitis	4 (22.2)	2 (16.7)	2 (33.3)
Panuveitis	12 (66.7)	8 (66.7)	4 (66.7)
Argyll-Robertson pupil	1 (5.6)	1 (8.3)	0 (0.0)
Acute syphilitic posterior placoid chorioretinitis	4 (22.2)	2 (16.7)	2 (33.3)
Optic neuritis	8 (44.4)	6 (50.0)	2 (33.3)

Baseline BCVA, intraocular pressure (IOP), anterior chamber cell grade, and anterior aqueous flare were also compared between patients who received topical and oral steroids (Table 4). We found there to be no statistically significant difference in BCVA, IOP, or anterior chamber cell grade/flare between the two groups at baseline.

**Table 4:** Similar baseline clinical characteristics topical versus oral steroid groups N: Number of eyes; SD: standard deviation; P-value from Student’s t-test

	Topical steroids mean ± SD N=12	Oral steroids mean ± SD N=6	P-value
Visual Acuity LogMAR (snellen equivalent)	0.96 ± 0.54 (20/200)	0.61 ± 0.60 (20/80)	0.2354
Intraocular pressure (mmHg)	14.25 ± 4.48	15.50 ± 6.22	0.6297
Anterior chamber cell grade	1.58 ± 1.29	2.00 ± 1.55	0.5541
Anterior aqueous flare	0.25 ± 0.62	0.00 ± 0.00	0.3464

Visual outcomes

**Figure 2:**
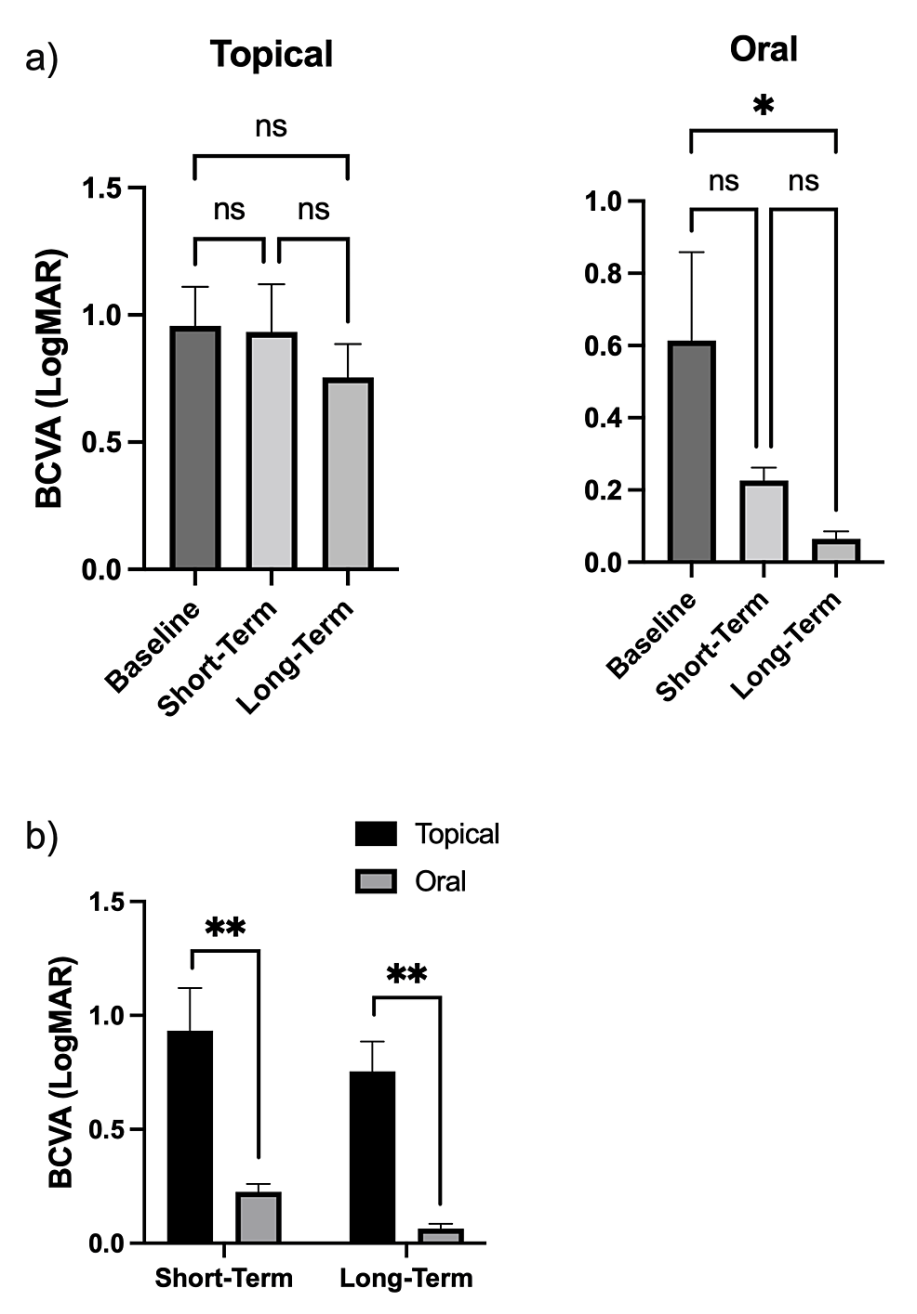
Best corrected visual acuity (BCVA) after topical or oral steroid treatment (a) BCVA (eyes) compared at baseline, short-term (<7 days) follow-up, and long-term (7 or more days) follow-up for eyes treated with topical steroid initiators or oral steroids. (b) BCVA (eyes) compared between oral and late steroid initiators at short-term and long-term follow-ups. n=6-12; ns p > 0.05, * p < 0.05, ** p < 0.01

Among eyes treated with topical steroids, BCVA improved from a mean logMAR (SD, Snellen fraction) of 0.96 (0.54, 20/200) to 0.93 (0.53, 20/170) at short-term follow-up, although this was not statistically significant (p=0.9946). At long-term follow-up, BCVA moderately improved further to 0.75 (0.46, 20/112) such that when comparing short-term to long-term follow-up, and baseline to long-term follow-up, no statistically significant differences were detected (p=0.7193 and 0.5945, respectively) (Figure 2a). Among eyes treated with oral steroids, BCVA improved from a mean logMAR (SD, Snellen fraction) of 0.61 (0.60, 20/80) at baseline to 0.23 (0.09, 20/110) at short-term follow-up (p=0.3882). At long-term follow-up, BCVA significantly improved from baseline to a mean LogMAR of 0.07 (.05, 20/25). When comparing BCVA at short-term follow-up to BCVA at long-term follow-up there was no statistically significant difference; however, BCVA from baseline to long-term follow-up demonstrated significant improvement (p=0.7129 and 0.0406, respectively) (Figure 2a). Absolute BCVA was compared between the topical and oral steroid groups at short-term and long-term follow-ups. At short-term follow-up, the mean BCVA of eyes in the topical steroid group at 0.93 (0.53, 20/170) was found to be significantly worse than the mean BCVA for the oral steroid group of 0.23 (0.09, 20/110) (p=0.0075). Similarly, the topical steroid group had a significantly worse BCVA at long-term follow-up of 0.75 (0.46, 20/112) compared to 0.07 (.05, 20/25) for the oral steroid group (p=0.0022) (Figure 2b).

BCVA at short- and long-term follow-ups were also compared among eyes that initiated adjunctive steroids simultaneously with antibiotic therapy (“early”) or after antibiotic therapy (“late”). No statistically significant differences were found between BCVA at short- and long-term follow-ups in either early or late steroid initiator groups. Among all eyes, 17 (94.4%) had an improved or stable BCVA at long-term follow-up. One eye (5.6%) from the topical steroid group worsened at long-term follow-up.

## Discussion

The current study evaluated the visual outcomes among patients treated with either oral or topical adjunctive steroids for ocular syphilis after treatment with IV penicillin. Although ocular syphilis is a known complication of syphilis infection that can meaningfully impact long-term visual acuity, the use of adjunctive steroids is not well-described or well-studied. Some clinicians have even feared that steroid use among patients with ocular syphilis may exacerbate the course of the disease. Notably, however, among the 18 eyes included in the current study, all but one eye had an improvement in BCVA or maintained BCVA at long-term follow-up when treated with either oral or topical steroids. In this study, at both short- and long-term follow-ups, eyes treated with oral steroids demonstrated improved BCVA when compared with eyes treated with topical steroids, indicating potentially superior visual outcomes with systemic steroid treatment.

Our findings are in line with several other studies which describe treatment protocols, clinical characteristics of ocular syphilis, and treatment outcomes. For example, a study by Gu et al. identified more than 8,000 cases of syphilis of which 213 patients had ocular syphilis representing 2.6% of their population [12]. In their study, they reported the treatment regimen and visual acuity outcomes for patients with ocular syphilis that met the WHO criteria for blindness. Similar to our study, they found that the most frequently prescribed antibiotic regimen was IV penicillin for a period of 14 days which was prescribed in 86% of patients. In contrast, the treatment regimen for steroids was largely variable across the population. Only 10 patients were treated with steroids: two received IV methylprednisolone followed by a taper of oral steroids, eight received oral steroids only, and no patients were treated with topical steroids. Notably, all 20 eyes among the 10 patients treated with steroids had either improvement or stabilization of visual acuity at follow-up. Similarly, we found that 17/18 eyes among the nine patients treated with steroids had improvement or stabilization of visual acuity at follow-up.

Moreover, a 2017 systematic review and meta-analysis by Zhang and colleagues described common clinical features of ocular syphilis and reviewed various treatment options including antibiotics and steroid use [4]. Overall, they reported a treatment success rate (defined as percentage of eyes with improved or maintained final VA after treatment) of 95% success rate for antibiotics and systemic steroids combined, similar to the success rate observed in the current study. Although eight studies in the review included topical steroid usage, success rates were not included in the results. Additionally, Zhang et al. reported that optic disc neuritis and panuveitis were the most consistent manifestations of ocular syphilis. In line with Zhang et al., we found that anterior chamber and vitreous chamber cells were the most common physical exam findings, indicative of uveitis manifestations, and that optic neuritis was also relatively common occurring in 44.4% of eyes in the sample. Rates of acute syphilitic posterior placoid chorioretinitis were comparable between the review (31.3%) and the current study (22.2%)

Zhang and colleagues also discussed possible risk factors associated with poor VA outcomes [4]. These identified risk factors included time between the onset of uveitis and treatment, longer duration of ocular symptoms, presence of macular edema or long-standing optic neuropathy, co-infection with HIV, and poor initial VA. Our study also analyzed the effect of time between penicillin and steroid initiation, though no statistically significant differences were seen in either treatment group at short- or long-term follow-up when stratified by initiation time of steroids. This is likely due to a type II error as our sample size consisted of 18 eyes and may not have been sufficiently powered to detect differences in the timepoint of steroid initiation. It is also worth noting that no patients in our study received steroids prior to the initiation of the penicillin as this could potentially exacerbate the infection and result in poor patient outcomes [13]. The patients in this study were also all immunocompetent with CD4+ counts higher than 500; therefore, more research needs to be conducted to assess the risk and benefits of initiating steroids in patients with low CD4+ counts.

Another study by Oliver and colleagues also reported the treatment preferences for syphilitic uveitis among a group of international ophthalmologists [14]. In this report, 103 uveitis specialists assessed their practices and provided details regarding the current approach to this disease. While a large proportion of group members indicated that they either routinely or sometimes prescribed adjunctive corticosteroid therapy, 70% indicated that they prescribed topical drops, 16% prescribed locally injected, and 82% prescribed systemic steroids. Initiation time of steroids also varied among members, with 17.1% initiating with antibiotic treatment, 54.9% initiating during but after the start of antibiotic treatment, and 4.9% initiating after antibiotic treatment was completed; 23.1% indicated that they did not have a standard timing of administration. This range of treatment prescriptions indicates a need for more prospective, controlled studies to help clinicians standardize treatment among syphilis patients with ocular manifestations.

Strengths and limitations

Strengths of the current study include its use of the real-world population from a mixed racial and urban-suburban population. Additionally, patients identified in this study followed up at several intervals after treatment was initiated, which enabled reporting of both short- and long-term treatment outcomes. The primary weakness of this study is the low sample size which consisted of nine patients (18 eyes). Because of our limited sample size, our study is susceptible to type II errors as our sample size may be underpowered to detect significant differences. The retrospective nature of the current study is also a limitation as it was difficult to control for confounding factors among the patient population, notably an unequal baseline BCVA between the topical and oral steroid groups. Further prospective studies are thus required to help confirm the results of this study.

## Conclusions

Though ocular syphilis is a known complication of secondary and tertiary syphilis infection, standardized treatment has not been established among ophthalmologists and empiric evidence of superiority between steroid routes has not been proven. The current study contributes to the current literature by comparing topical steroids to oral steroids with promising results regarding visual acuity outcomes. Early identification of patients with ocular syphilis and timely referral for treatment with penicillin and adjunctive steroids is crucial in preventing disease progression and improving visual outcomes. Due to the small size and retrospective nature of the study, further prospective studies will be required to confirm these results.
